# Regulation of sensory perception and motor abilities by brain-specific action of chromatin remodeling factor CHD1

**DOI:** 10.3389/fnmol.2022.840966

**Published:** 2022-08-02

**Authors:** Ines Schoberleitner, Birte Mertens, Ingo Bauer, Alexandra Lusser

**Affiliations:** Institute of Molecular Biology, Biocenter, Medical University of Innsbruck, Innsbruck, Austria

**Keywords:** chromatin remodeling factor, neuron, perception, olfaction, locomotion, transcriptional regulation, histone variant

## Abstract

The ATP-dependent chromatin remodeling factor CHD1 (chromodomain-helicase-DNA binding protein 1) is involved in both the *de novo* assembly and the remodeling of chromatin. Recently, we discovered a crucial role of CHD1 in the incorporation of the histone variant H3.3 in the fly brain illustrated by widespread transcriptional upregulation and shortened lifespan in *Chd1*-mutant animals. Because many genes linked to sensory perception were dysregulated in *Chd1*-mutant heads, we studied the role of CHD1 in these processes. Here we show that *Chd1*-mutant flies have severe defects in their response behavior to olfactory and gustatory but not visual stimuli. Further analyses suggested that poor performance in gustatory response assays was caused by reduced motivation for foraging and feeding rather than defects in taste perception. Moreover, we show that shortened lifespan of *Chd1*-mutant flies is accompanied by indications of premature functional aging as suggested by defects in negative geotaxis and exploratory walking assays. The latter phenotype was rescued by neuronal re-expression of *Chd1*, while the olfactory defects were not. Interestingly, we found evidence for indirect regulation of the non-neuronal expression of odorant binding proteins (*Obp*) by neuronal expression of *Chd1*. Together, these results emphasize the crucial role of CHD1 activity controlling diverse neuronal processes thereby affecting healthy lifespan.

## Introduction

The SWI/SNF family member CHD1 (chromodomain-helicase-DNA binding protein 1) is an ATP-dependent chromatin remodeling and assembly factor (Marfella and Imbalzano, 2007; Clapier and Cairns, 2009). Since a major function of chromatin remodeling factors is to effect changes of histone-DNA interactions within or between nucleosomes, they are critical components of all processes that require access to the DNA substrate, most prominently transcription, replication, and DNA damage repair. Together with histone chaperones, such as NAP1, CHD1 can facilitate *in vitro* the assembly of nucleosomes into regularly spaced arrays (Lusser et al., 2005). *In vivo*, it has diverse roles that include functions in transcription regulation, transcription-independent chromatin assembly as well as in DNA damage repair (Simic et al., 2003; Konev et al., 2007; Simsiii et al., 2007; Petesch and Lis, 2008; Srinivasan et al., 2008; Gaspar-Maia et al., 2009; Skene et al., 2009; Morettini et al., 2011; Guzman-Ayala et al., 2015; Siggens et al., 2015; de Dieuleveult et al., 2016; Kari et al., 2016; Lee et al., 2017; Rüthemann et al., 2017; Shenoy et al., 2017).

A transcription- and replication-independent role of CHD1 is the assembly of histone H3.3 into paternal pronuclear chromatin in *Drosophila*, which is achieved in concert with the histone chaperone HIRA (Loppin et al., 2005; Konev et al., 2007). The H3.3 variant is considered a “replacement”-type histone, because it is deposited in a replication-independent manner, for instance during nucleosome re-assembly in the wake of transcribing polymerases (Talbert and Henikoff, 2010). Recently, we have also implicated CHD1 in H3.3 assembly in the fly brain. Its deletion resulted in reduced chromatin-associated H3.3 levels and widespread upregulation of transcription. Moreover, sugar, fatty acid, and amino acid metabolism was severely impaired, and *Chd1*-mutant flies exhibited reduced food intake and a strong shortening of lifespan. Consistent with this, we found that many genes linked to the regulation of hunger and satiety were dysregulated in the heads of mutant flies. Re-expression of CHD1 only in neurons rescued all these phenotypes (Schoberleitner et al., 2021) emphasizing the critical role of this chromatin assembly factor for brain function.

Food intake requires the recognition of appropriate food, which is to a large part conferred by the ability to taste and smell and also requires vision to evaluate and discriminate sumptuous food sources from dangerous substances in the environment. Because we found genes linked to sensory perception to be enriched among the genes dysregulated in *Chd1*-mutant flies, we set out to investigate the contribution of CHD1 to sensory perception and locomotory behavior. The results revealed that CHD1 is not required for the response to light, but that its absence causes impaired gustatory and olfactory response as well as compromised locomotory behavior. Furthermore, we found that the lack of response to the repelling odorant benzaldehyde was neither dependent on *Chd1* expression in neurons, nor was it caused by the dysregulation of odorant binding proteins (*Obp*) observed in *Chd1*-mutant heads suggesting that functions in other cell types might be responsible for the olfactory defect.

## Materials and methods

### *Drosophila* strains and husbandry

Fly stocks were maintained at 25°C and 60% humidity in a 12/12 h light/dark cycle in batches of 20 flies on sugar-cornmeal media as described previously (Sebald et al., 2016). All mutations and transgenes were studied in a *w*^1118^ background. *Chd1* deficient (*Chd1^–/–^*) flies were obtained by crossing *Df(2L)Chd1^1^/CyO* with *Df(2L)Exel^7014^/CyO*. *Chd1^WT/WT^* flies were obtained from crosses of *Df(2L)Chd1^1^,P{Chd1*^WT^*}/CyO* and *Df(2L)Exel^7014^,P{Chd1*^WT^*}/CyO*. For pan neural induction of *Chd1* in *Chd1* mutant flies (*Chd1^–/–^*; *elav-Gal4*>*UAS-Chd1*^WT^**; termed *Chd1^elav^*), the lines *Df(2L)Chd1^1^,P{UASt-Chd1*^WT^*}/CyO* and *Df(2L)Exel^7014^/CyO; elav-Gal4/TM3* were combined. *Elav-Gal4* (Stock ID 8760) line was obtained from the Bloomington Stock Center. For detailed description of strain genotypes see Schoberleitner et al. (2021).

### Behavior and perception experiments

For all assays described below, batches of female virgin flies in 3 or 5 technical replicates (20 flies each) at different ages were tested at the same time of day (10 a.m.) in a uniformly illuminated area at constant temperature unless otherwise stated.

#### Phototaxis assay

To test the response to visual light we followed a procedure by Vang et al. (2012). Briefly, flies were starved in vials containing 1.5% agar for 18 h. Then they were transferred to an empty vial by tapping, and the vial was connected to a 25 cm long test tube in a dark room 30 min prior to the test to adapt to darkness. The horizontal test tube was then gently pounded down to position the flies at one end, away from the light source. A perpendicular light source (15 cm distance) was placed at the other end of the test tube (Figure 2A). To start the test, the cold light source (Schott KL 1500 LCD, 15V, 150W, 3300K, position 5) was switched on thereby establishing a light gradient of about 90 lux at the nearest point to about 3 lux at the furthest point. Every minute in the 10 min test duration, the number of flies in each third of the test tube was scored. Data were expressed as attraction response index (RI%) corresponding to the percentage of flies in the segment closest to the light source relative to the total number of flies placed in the test tube. The test was repeated three times and data were plotted as mean ± SEM.

#### Olfactory chemotaxis assay

Response to volatile repellents was assayed as described in Vang et al. (2012). Briefly, flies were starved in vials containing 1.5% agar for 18 h before transferring them to an empty test vial 30 min prior to the test. To start the test, the vial was connected to a test tube containing 100 mM or 10 mM benzaldehyde in 1.5% agar at one end and the flies were gently tapped toward the repellent (Figure 2C). The number of flies in each third of the test tube was scored every minute for 10 min. Data were expressed as repulsion response index (RI%), corresponding to the percentage of flies in the segment furthest from the attractant relative to the total number of flies placed in the test tube. The test was repeated three times and data were plotted as mean ± SEM.

#### Gustatory chemotaxis assay

Response to non-volatile chemicals was tested as described by Vang et al. (2012). Briefly, flies were starved in vials containing 1.5% agar for 18 h before transferring them by tapping to an empty test vial 30 min prior to the test. The test was started by connecting the vial with a 25 cm long test tube containing 100 mM sucrose in 1.5% agar at one end and gently tapping the flies to the end of the empty vial (Figure 2E). Every minute for 10 min the number of flies in each third of the test tube was scored. Data were expressed as attraction response index (RI%), corresponding to the percentage of flies in the segment closest to the attractant relative to the total number of flies placed in the test tube. The test was repeated three times and data were plotted as mean ± SEM.

#### Proboscis extension reflex assay

To evaluate the flies’ ability to display reflex-like response to attractive substances in the food and motivation to feed, we assayed the proboscis extension reflex. Thirty starved (24 h) female virgin flies of each genotype were presented with glass capillaries containing a solution of sucrose to the proboscis by making contact, and the number of proboscis extension responses was determined as described in Shiraiwa and Carlson (2007) and Qi et al. (2015). Presentation of stimulus was repeated in 10 trials per fly (*n* = 30/genotype). The experiment was performed once, and data were plotted as mean ± SD.

#### Negative geotaxis assay

The startle-induced negative geotaxis assay was performed as described in Ismail et al. (2015) (Figure 3A). Flies of the indicated ages were transferred to climbing vials (two empty vials connected face to face) without anesthesia and left to acclimatize for 1 h. Testing was performed by tapping down the flies and counting the number of flies that climb past a mark on the vials (8 cm from the bottom) within 60 sec using a custom-made apparatus. In each experiment, the test was repeated 10 times (trials) with 1 min breaks in between. The results were analyzed as% of climbing flies at each time point (*n* = 5 batches of 10–12 flies/genotype/experiment). The experiment was repeated three times and data were plotted as mean ± SEM.

#### Exploratory walking assays

To assess walking behavior the exploratory walking assay was performed as previously described by Ismail et al. (2015) (Figure 3C). Briefly, female virgin flies were placed individually into the center of a 14.5 cm petri dish with a 1 cm square grid. The number of grid-line crossings during 1 min was scored and graphically presented (*n* > 40/genotype/experiment). The experiment was repeated three times and data were plotted as mean ± SEM.

### RNA-seq data source

RNA-seq data from virgin female *Drosophila* heads (Schoberleitner et al., 2021); GEO accession number (GSE146392) were subjected to gene ontology analysis using the GOrilla tool (Eden et al., 2009) by comparing the unranked target list (differentially expressed genes with log2 fold change ≥1, adjusted *p*-value ≤ 0.05 and base mean ≥20) with a background list containing all transcripts identified in the sequencing analysis. Heatmaps of significantly enriched gene categories were generated using the R package pheatmap (Kolde, 2019).

### Statistics

Graphing and statistics were performed using GraphPad Prism software v.8.2.1. The level for statistical significance was set at *p* ≤ 0.05 for all statistical tests and significant differences were marked (*p ≤ 0.05, ^**^p ≤ 0.01, ^***^p ≤ 0.001, ^****^p ≤ 0.0001, ns, not significant).

#### Sensory perception (chemotaxis, phototaxis, and proboscis extension reflex)

Data from photo- and chemotaxis experiments were quantified and interpreted as response toward the non-volatile and volatile chemicals as well as light. To calculate the statistical significance of the experimental clustering (interaction trial × age × genotype effect), 3-way ANOVA analysis based on distance matrices (distances within all replicates versus distances between all replicates) was performed. Data from proboscis extension reflex (PER) experiments were presented as response frequency, i.e., percentage of responding flies per stimulation trial (total of 10) (Figure 2H). The experiment was performed once (*n* = 30/genotype) for each age (4 and 14 days). To estimate the statistical significance of the response frequency clustering (stimulation trial × genotype effect), 2-way ANOVA analysis based on distance matrices (distances within all replicates versus distances between all replicates) for each age timepoint (4 and 14 days) was performed.

#### Mobility assays

Results obtained from the negative geotaxis assay were illustrated as climbing capability, and statistical analysis of differences between the two fly lines at the respective age and each given sequence cycle was performed by 3-way ANOVA based on distance matrices (distances within all replicates versus distances between all replicates). Exploratory walking behavior was scored as number of grid-line crossings per minute, and all data are graphically presented as individual values (*n* > 40/genotype/experiment, three experiments). Statistical analysis of explorative walking differences between the two fly lines at the respective age was performed by unpaired Student’s *t*-test.

## Results

### Absence of chromodomain-helicase-DNA binding protein 1 causes dysregulation of sensory perception genes

**FIGURE 1:**
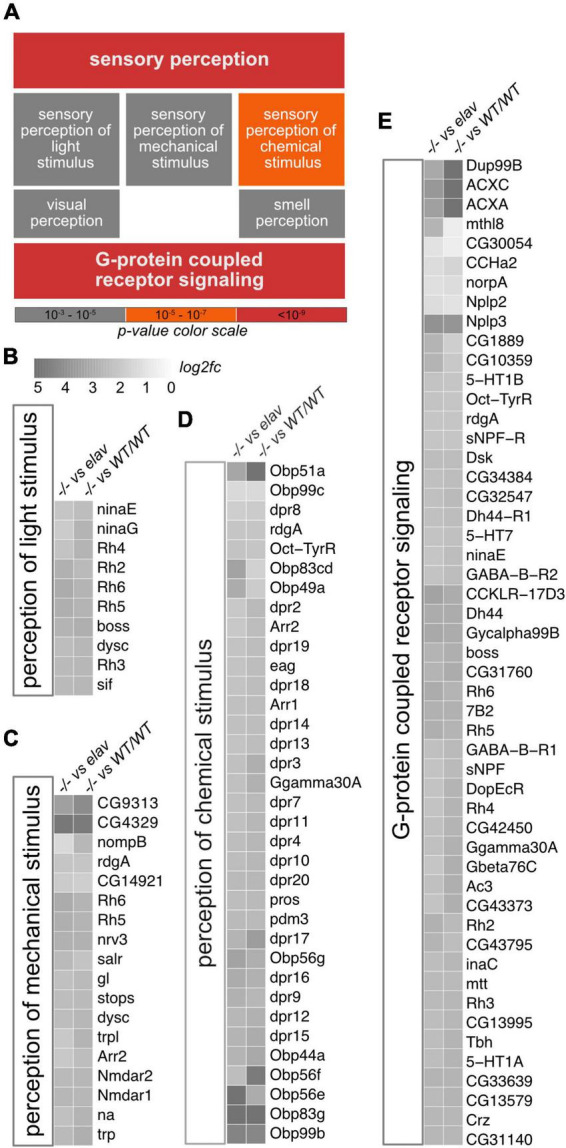
Gene ontology (GO) enrichment analysis of upregulated genes in *Chd1^–/–^* heads. **(A)** Significantly enriched GO terms from the analysis of 1897 genes that were upregulated in *Chd1^–/–^* versus *Chd1^WT/WT^* as well as *Chd1^–/–^* versus *Chd1^elav^* (log2 fold change ≥1, adjusted *p* ≤ 0.05, base mean ≥20). Color code signifies significance of enrichment as indicated. **(B–E)** Heatmaps of differentially regulated genes in enriched categories. Color scale in panel **B**, indicating log2 fold change (*log2fc*), applies to all subpanels.

To examine if and how CHD1 might affect sensory perception in *Drosophila*, we turned to our previously generated RNA-seq data from fly heads (Schoberleitner et al., 2021). The data set contains gene expression profiles from *Chd1*-deletion mutant flies (termed *Chd1^–/–^*), from *Chd1*-deletion mutant flies rescued by transgenic expression of *Chd1* under the control of its native promoter (termed *Chd1^WT/WT^*) and from mutant flies that were rescued by neuron-specific expression of *Chd1* under the control of the *elav* promoter (termed *Chd1^elav^*). We intersected the expression profiles of these lines and performed gene ontology (GO) term enrichment analysis with the 1,878 genes that were upregulated in *Chd1^–/–^* compared to *Chd1^WT/WT^* and *Chd1^elav^* fly heads. Among the highly enriched GO categories were genes associated with sensory perception including sensory perception of light, mechanical and chemical stimuli (Figure 1A). For instance, *rhodopsin* genes, genes encoding *Obp* and *defective proboscis extension response* (*dpr*) genes as well as genes encoding N-methyl-D-aspartate (NMDA) receptors were significantly upregulated in *Chd1^–/–^* flies compared to *Chd1^WT/WT^* or *Chd1^elav^* (Figures 1B–D). Furthermore, many G-protein coupled signaling-linked genes had increased transcription in *Chd1^–/–^* compared to *Chd1^WT/WT^* or *Chd1^elav^* heads (Figure 1E; Schoberleitner et al., 2021). These included genes related to serotonin-, dopamine-, GABA-, or octopaminergic signaling, along with allostatic, acetylcholine, rhodopsin, and tachykinin associated signaling (Figure 1E). The dysregulation of these genes suggested that sensory abilities of *Chd1*-deficient flies might be compromised.

**FIGURE 2:**
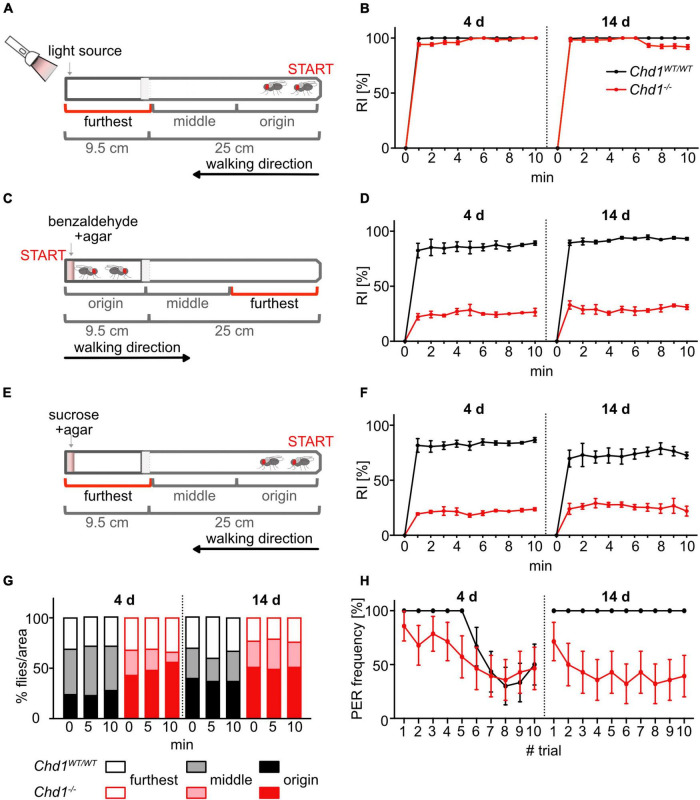
Absence of CHD1 impairs olfactory and gustatory perception. **(A)** Experimental set-up of the assay used to assess positive phototaxis. **(B)** Positive phototaxis response indices (RI) of 4 and 14 days old *Chd1^WT/WT^* and *Chd1^–/–^* flies. Mean ± SEM of six replicates (20 flies per group) are shown. **(C,E)** Experimental set-up to test olfactory behavior in response to the repellent odorant benzaldehyde **(C)** and gustatory response behavior to sucrose **(E)**. **(D,F)** Repulsion **(D)** and attraction **(F)** response indices of 4 and 14 days old *Chd1^WT/WT^* and *Chd1^–/–^* flies. Mean ± SEM of six technical replicates (20 flies per group) and three independent experiments are shown. 3-way ANOVA of **(B,D,F)**: genotype main effect: **(B)** *F*(1,8) = 124.66, *p* < 0.0001; **(D)** *F*(1,88) = 3809, *p* < 0.0001; **(F)** *F*(1,88) = 1680, *p* < 0.0001; age main effect: **(B)** *F*(1,8) = 3.267, ns; **(D)** *F*(1,88) = 28.41, *p* < 0.0001; **(F)** *F*(1,88) = 3.500, *p* = 0.0647; trial main effect: **(B)** *F*(10,80) = 5872, *p* < 0.0001; **(D)** *F*(10,88) = 135.8, *p* < 0.0001, **(F)** *F*(10,88) = 58.59, *p* < 0.0001; interaction (age × genotype) effect: **(B)** *F*(1,8) = 3.474, *p* = 0.0993; **(D)** *F*(1,88) = 1.289, *p* = 0.2594; **(F)** *F*(1,88) = 28.10, *p* < 0.0001; interaction (trial × age × genotype) effect: **(B)** *F*(10,80) = 4.458, *p* < 0.0001; **(D)** *F*(10,88) = 0.3576, *p* = 0.9613; **(F)** *F*(10,88) = 0.34789, *p* = 0.8996, **(G)** Behavior of flies in the test set-up shown in panels **C,E** in the absence of any stimulus. Percentage of flies in the different sectors at 0, 5, and 10 min of the test. Mean ± SEM of 3 technical replicates (20 flies per group) and 3 independent experiments are shown. 2-way ANOVA of interaction (genotype × time lapse) effect: *F*(22,684) = 887713; genotype main effect: *F*(11,684) = 517.0; time lapse effect: *F*(2,684) = 2934590; age main effect: *F*(9,27) = 3.535, *p* = 0.0052; genotype main effect: *F*(3,27) = 28.22. **(H)** Proboscis extension response (PER) frequency in 10 sequential tastant offerings with ingestion permitted (*n* = 30 per genotype and age). 2-way ANOVA of age main effect: *F*(9,27) = 3.535; genotype main effect: *F*(3,27) = 28.22.

**FIGURE 3:**
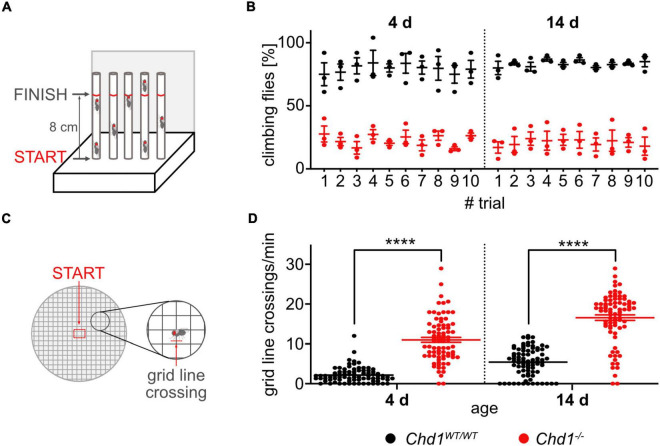
Compromised climbing and exploratory walking behavior of *Chd1*-deficient flies. **(A)** Experimental set-up of the negative geotaxis assay. **(B)** Percentage of 4 and 14 days old flies climbing to 8 cm height within 60 s in 10 trials. Mean ± SEM of 5 cohorts of 10–12 flies per genotype from 3 independent experiments is shown. 3-way ANOVA results for genotype main effect: *F*(1,8) = 197.2, *p* < 0.0001; age main effect: *F*(1,8) = 0.05742, *p* = 0.8167. **(C)** Experimental set-up for exploratory walking behavior. **(D)** Gridline crossings of individual 4 and 14 days old flies during 1 min were scored. Three independent experiments of *n* > 40 per genotype/experiment were performed. Statistical significance was determined by unpaired Student’s *t*-test. ****p < 0.0001.

#### Chromodomain-helicase-DNA binding protein 1 is required for sensory perception in *Drosophila*

To examine this idea, we performed various experiments to assess the behavioral response of the flies to sensory cues. We first tested response to light using a simple phototaxis assay (Vang et al., 2012). Mated female flies (4 or 14 days old) were placed in a 34 cm long tube, gently tapped to the bottom of the vial and scored for their tendency to move toward a light source that was placed at the opposite end of the tube (Figure 2A). The number of flies present in each area of the test tube, i.e., at the origin, in the middle and the furthest part (Figure 2A), was counted every minute. Interestingly, the experiment revealed no difference between *Chd1^–/–^* and *Chd1^WT/WT^* (Figure 2B). Young *w*^1118^ flies (wildtype background control) also responded to the light by immediately walking toward it, but then exhibited a tendency to move backward again accounting for the slightly lower RI values detected with this line (Supplementary Figure 1A). Thus, *Chd1* deletion did not affect light reception and positive phototaxis behavior, although we cannot rule out that other aspects of vision are compromised.

Next, we tested olfactory abilities by observing the behavior of *Chd1-*deficient and *Chd1-*wildtype flies in a similar experimental set-up, except that the strongly repellent odor benzaldehyde was added to an agar disc that was placed at one end of the apparatus (Figure 2C). As expected, wildtype flies (*w*^1118^, *Canton S*) and *Chd1^WT/WT^* flies at both ages showed strong repulsion by benzaldehyde by walking away from the repellent after exposure (Figure 2D and Supplementary Figure 1B). By contrast, about 70% of 4- and 14-day-old *Chd1*-deficient flies remained close to the benzaldehyde source, where they had been placed at the start of the assay (Figure 2D). Because *Chd1^–/–^* flies showed similar walking behavior in the phototaxis assay as the wildtype flies, it is not likely that a potential impairment of locomotory abilities acted as a confounding parameter in this assay. Thus, the results suggest that absence of CHD1 strongly impacts on the sense of smell.

To assess the gustatory abilities of the flies, sucrose was offered as an attractant in the assay set-up shown in Figure 2E. Sucrose must be tasted because it is not volatile. Starved *Chd1^WT/WT^* flies of both ages departed from the non-sucrose end, and the majority accumulated in the compartment nearest to the sucrose similar to *w*^1118^ flies (Figure 2F and Supplementary Figure 1C). By contrast, the behavior of *Chd1^–/–^* flies was comparable to the smell test with only about 20.2 ± 0.3% of the 4-day-old and 27.9 ± 0.5% of the 14-day-old mutant flies moving close to the sucrose source (Figure 2F). To ascertain that the observed aberrant behavior of *Chd1*-deficient flies was caused by defective gustatory perception and not by reduced motivation for foraging, we subjected the flies to the same assay set-up yet omitting the taste stimulus. The 4 days old starved *Chd1^WT/WT^* flies showed roughly equal distribution across all sections of the tube at each time point with a slight preference for the middle part, whereas older wildtype flies slightly preferred the origin and furthest sections. *Chd1^–/–^* flies were similar to the older wildtype flies except for a slight preference of the origin section at both ages (Figure 2G) suggesting that reduced motivation to forage might indeed contribute to the poor gustatory response observed in the tube assay. To examine taste abilities of the flies by another method, we employed the PER assay (Shiraiwa and Carlson, 2007). When the taste neuron-innervated sensilla of the fly’s labellum make contact to an attractive substance, such as sucrose solution, the proboscis is extended to consume the food. Monitoring the frequency of PER across 10 experimental trials, we observed that in young flies, the PER frequency progressively decreased in both *Chd1^WT/WT^* and *Chd1^–/–^* flies most likely reflecting progressive satiety (Wang et al., 2004; Shiraiwa and Carlson, 2007; Slone et al., 2007; Chen and Amrein, 2014). At 14 days of age, *Chd1*-wildtype flies showed 100% response frequency throughout the experiment, whereas *Chd1*-mutant flies started with about 75% response which gradually decreased to about 50% response (Figure 2H). Given that young *Chd1*-deficient flies exhibited almost normal PER, it is likely that their gustatory abilities are principally intact, at least at this young age. At 14 days of age, most of the flies initially responded to the stimulus but very quickly turned to satiety-like behavior. Together, the results from the gustatory tube assay and the PER assay suggest that absence of CHD1 does not cause a (complete) loss of taste perception. Rather, it appears that the flies can still taste but might have diminished motivation to feed. This is consistent with our previous observations that *Chd1*-deficient flies show strongly reduced food intake correlating with dysregulation of many neuropeptide genes responsible for feeding and foraging behavior (Schoberleitner et al., 2021).

#### Deficient negative geotaxis and exploratory walking behavior: Indicators of neurodegenerative alterations in *Chd1*-deficient flies?

Although walking abilities of *Chd1^–/–^* flies appeared similar to wildtype flies in the phototaxis assay described above, we performed two additional locomotory assays. The negative geotaxis assay measures the startle-induced reflexive tendency of flies to move against gravity (Figure 3A), while in the exploratory walking assay (Figure 3C), more complex behavior involving the regulation of walking parameters, such as speed and direction, as well as orientation is assessed. Both, reflexive as well as decision-based locomotion, however, are substantially affected by age-related decline and are therefore also used to test for functional senescence (Grotewiel et al., 2005; Ismail et al., 2015).

We found that both 4 and 14 days old *Chd1^–/–^* flies performed significantly worse in the negative geotaxis assay compared to wildtype (Figure 3B and Supplementary Figure 1D). *Chd1*-deficient flies were slow climbing upward and showed numerous direction changes compared to *Chd1-*wildtype and *w*^1118^ flies that were moving only upward.

For the exploratory walking assay, flies were placed individually into the middle of a graduated petri dish, and their movement was monitored by counting the number of grid-line crossings (Figure 3D). Typically, the physical borders of an open field (borders of the Petri dish) are of main interest for the fly, and it will normally strive to head there (Soibam et al., 2012). Accordingly, *Chd1*-wildtype flies after placement into the center of the dish either remained rooted to the spot observing the environment for a short time or walked by the shortest path toward the border of the Petri dish and remained there. A similar, albeit not as pronounced behavior was displayed by *w*^1118^ flies (Supplementary Figure 1E). By contrast, *Chd1-*mutant flies immediately started to walk in circles (many grid-line crossings) but did not head for the border (Figure 3D). When 14 days old flies were tested, we observed that the tendency to go to the border of the dish by the shortest way decreased also for *Chd1*-wildtype flies, but the difference between the genotypes remained (Figure 3D). The reduced performance of the flies in these test paradigms in the absence of CHD1 suggest an earlier entry into a state of age-related decline compared to wildtype flies.

#### Brain-specific expression of *Chd1* rescues most but not all sensory defects in mutant flies

The RNA-seq data had shown that most dysregulated genes in *Chd1^–/–^* heads were rescued by both neuron-specific expression of *Chd1* or expression under the control of its native promoter (Schoberleitner et al., 2021). To examine, if neuronal expression of *Chd1* in the *Chd1*-deficient background would affect the sensory and locomotory abilities of the fly, we performed negative geotaxis, exploratory walking, gustatory, and olfactory assays also with *Chd1^elav^* flies.

**FIGURE 4:**
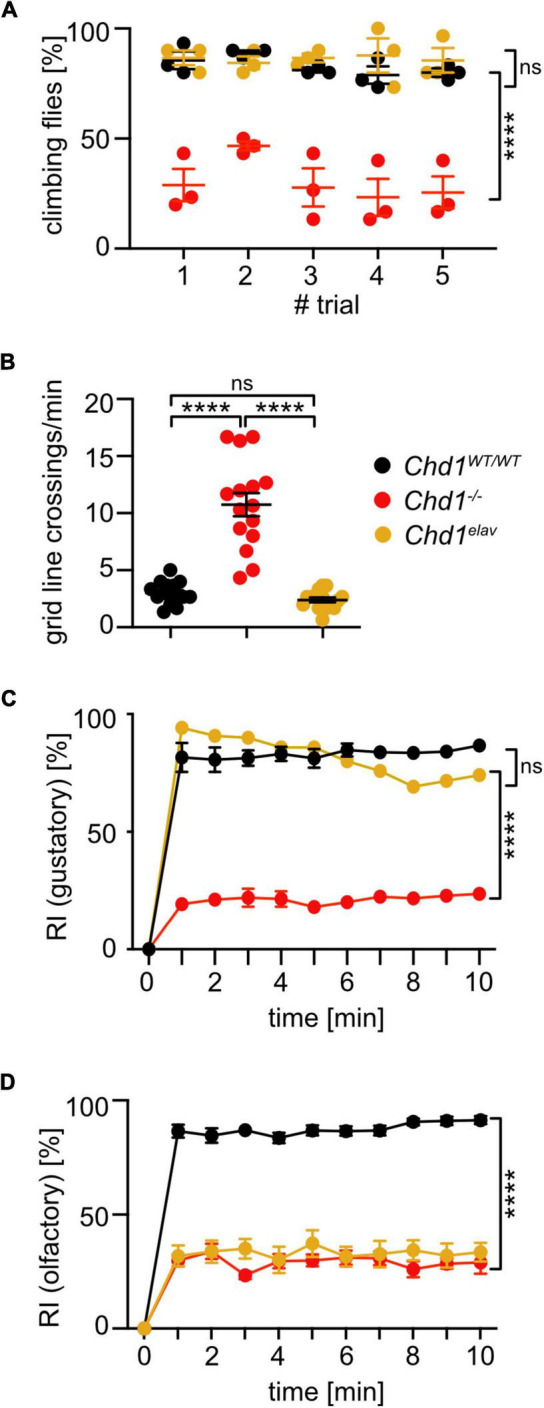
Pan-neuronal expression of *Chd1* rescues locomotory and gustatory impairments but has no effect on olfactory response behavior. **(A)** Negative geotaxis assay to assess startle-induced climbing behavior. Percentage of 7 days old flies climbing to 8 cm height within 60 s was determined in 5 trials. Mean ± SEM of 3 cohorts of 10 flies each per genotype/experiment from 3 independent experiments is shown. Significant differences between fly lines was determined by unpaired Student’s *t*-test. ****p < 0.0001, ns, not significant. **(B)** Gridline crossings in exploratory walking assay of individual 7 days old flies during 1 min were scored. Three independent experiments of *n* > 15 per genotype/experiment were performed. Statistical significance was determined by unpaired Student’s *t*-test. ****p < 0.0001. **(C)** Attraction response index (RI) to sucrose of 7 days old flies was tested as described in Figure 2. Mean ± SEM of three technical replicates (20 flies per group) and three independent experiments are shown, except for *Chd1^elav^* flies which were tested in 6 technical replicates (20 flies each) and one experiment. **(D)** Benzaldehyde of 7 days old flies was tested as described in Figure 2. Mean ± SEM of three technical replicates (20 flies per group) and three independent experiments are shown. Statistical significance was determined by 2-way ANOVA: Genotype main effect: **(C)** *F*(2,44) = 44.46, ****p < 0.0001, and **(D)** *F*(7,704) = 457.6, ****p < 0.0001; trial main effect: **(C)** *F*(10,44) = 254.4, ****p < 0.0001, and **(D)** *F*(10,704) = 110.4, ****p < 0.0001; trial × genotype interaction effect: **(C)** *F*(20,44) = 3910, ****p < 0.0001, and **(D)** *F*(70,704) = 5.453, ****p < 0.0001.

The results show that negative geotaxis, exploratory walking abilities, and gustatory response were completely rescued by expression of *Chd1* with the *elav* driver (Figures 4A–C) indicating that activity of CHD1 in neurons is responsible for the respective defects in *Chd1^–/–^* flies. Intriguingly, however, neuronal expression of *Chd1* was not sufficient to rescue the olfactory deficits of *Chd1^–/–^* flies (Figure 4D). Hence, for proper olfactory response, CHD1 activity in other cell types in addition to neurons is required.

## Discussion

We have previously shown that CHD1 expression in the brain is required for normal regulation of several metabolic parameters, such as sugar, fatty acid, and amino acid homeostasis. Lack of CHD1 results in reduced food intake, increased global inflammation and premature death. On the molecular level, these phenotypes are linked to reduced chromatin-associated levels of the histone variant H3.3, which is incorporated into chromatin mostly during transcription-related processes that lead to the loss of histones. Consequently, *Chd1^–/–^* flies exhibit increased global transcription presumably due to a general relief of chromatin-mediated repression (Schoberleitner et al., 2021). Here we expand on these findings and show that absence of CHD1 results in olfaction deficits, reduced gustatory response, which is most likely caused by reduced motivation for feeding, as well as age-related locomotory impairments. GO enrichment analyses revealed that these phenotypic deficits are in agreement with the dysregulation of genes known to be involved in these processes. Re-expression of *Chd1* in neurons rescued the expression of most of the dysregulated genes (Figure 1) as well as the climbing, exploratory walking and gustatory abilities of the flies. Given that the two locomotion assays are often used to assess functional aging (Grotewiel et al., 2005; Ismail et al., 2015), these results, in combination with our previous findings revealing that neuronal expression of *Chd1* rescues shortened lifespan and chronic inflammation, support the notion that CHD1 plays an important role in the prevention of premature aging.

The reasons for the complete failure to restore olfactory perception by neuronal *Chd1* expression, however, remain unknown. Deletion of *Chd1* caused the upregulation of 16 out of 34 *Obp* genes that were detected in our RNA-seq analysis suggesting an apparently obvious mechanism to explain the olfactory response phenotype. However, even though transcription of almost all dysregulated *Obp* genes was restored in *Chd1^elav^* flies, olfactory response was still impaired. Diverse functions have been ascribed to OBPs in insects, ranging from roles in the transport of odorants to odorant receptors, the sequestration of odorants from the sensillum lymph, the function as co-ligands at neuronal receptors or the protection of odorants from degradation (Scheuermann and Smith, 2019). Even though most studies have examined the functional effects of OBP ablation rather than of overexpression as we observe here, reduction of certain OBPs sometimes resulted in increased response to certain odorants whereas response to others was lost indicating that the mode of action of OBPs is complex and may be different for different OBPs, different odorants and different cell types (Swarup et al., 2011; He et al., 2019; Scheuermann and Smith, 2019). Interestingly, a recent study reported that deletion of the ten most abundant *Obp* genes in antennal basiconic sensilla did not affect olfactory abilities of the flies (Xiao et al., 2019) leading the authors to conclude that olfaction is not dependent on the abundant OBPs or that minor OBPs can compensate (Xiao et al., 2019). Our results would support this notion in the sense that the dysregulation/rescue of *Obp* expression had no effect on the flies’ olfactory response, at least the response to benzaldehyde. It is possible, however, that the reactions are different for other odorant types. Regardless of the potential role of OBPs in the olfaction process, our results also suggest that functions of CHD1 outside of neurons must be critical for the failure of *Chd1^elav^* flies to respond to benzaldehyde.

Another intriguing observation from our study is that neuronal expression of *Chd1* is sufficient to restore expression of *Obp* genes. OBPs are synthesized by non-neuronal chemosensory support cells in antennal and other sensilla and secreted into the lymph of the sensillum. Thus, the rescue of *Chd1* expression in *Chd1^elav^* flies, which is supposed to occur only in the sensory neurons, actually should not affect the regulation of *Obp* genes in the support cells. A hint toward a potential mechanism to explain this apparent discrepancy comes from a recent study on the function of *Obp69a* in translating social interaction into sex-specific behavior involving the male-specific pheromone 11-cis-Vaccenyl acetate (cVA). The authors found that expression of *Obp69a* in the auxiliary cells was regulated by active neurotransmission of the cVA sensing neuron to the second order olfactory neuron (Bentzur et al., 2018). In analogy to this, we speculate that the activity of sensory neurons in *Chd1^–/–^* flies might be altered, which is also illustrated by the dysregulation of multiple genes linked to G-protein-coupled receptor signaling and neurotransmission (Figure 1E; Schoberleitner et al., 2021). This may affect the regulation of *Obp* genes in the support cells. Upon neuronal re-expression of *Chd1* (*Chd1^elav^*), neuronal function is restored (Figure 1E), which may cause the downregulation of the *Obp* genes in the support cells.

In summary, we have characterized CHD1 as an important factor contributing to neuronal function and regulating sensory and locomotory behavior. As a chromatin regulator, the molecular mechanism behind these functions involves regulation of transcription including, but not limited to, histone variant H3.3 incorporation thereby maintaining chromatin integrity (Schoberleitner et al., 2021). Considering that CHD1 as well as its significance for brain function are conserved between insects and mammals (Piatti et al., 2015; Schoberleitner et al., 2019; Cardoso et al., 2021), these findings may also be relevant for the study of age-related decline processes in humans.

## Data availability statement

The datasets presented in this study can be found in online repositories. The names of the repository/repositories and accession number(s) can be found in the article/Supplementary material.

## Author contributions

IS and AL conceived the project, designed the experiments, and wrote the manuscript. IS and BM performed experiments and analyzed the data. IB analyzed the RNA-seq data. All authors read and approved the final manuscript.

## References

[B1] Bentzur A. Shmueli A. Omesi L. Ryvkin J. Knapp J. M. Parnas M. (2018). Odorant binding protein 69a connects social interaction to modulation of social responsiveness in *Drosophila*. *PLoS Genet.* 14:e1007328. 10.1371/journal.pgen.1007328 29630598 PMC5908198

[B2] Cardoso A. R. Lopes-Marques M. Oliveira M. Amorim A. Prata M. J. Azevedo L. (2021). Genetic variability of the functional domains of chromodomains helicase DNA-Binding (CHD) proteins. *Genes (Basel)* 12:1827. 10.3390/genes12111827 34828433 PMC8623811

[B3] Chen Y. Amrein H. (2014). Enhancing perception of contaminated food through acid-mediated modulation of taste neuron responses. *Curr. Biol.* 24 1969–1977. 10.1016/j.cub.2014.07.069 25131671 PMC4332783

[B4] Clapier C. R. Cairns B. R. (2009). The biology of chromatin remodeling complexes. *Annu. Rev. Biochem.* 78 273–304. 10.1146/annurev.biochem.77.062706.153223 19355820

[B5] de Dieuleveult M. Yen K. Hmitou I. Depaux A. Boussouar F. Bou Dargham D. (2016). Genome-wide nucleosome specificity and function of chromatin remodellers in ES cells. *Nature* 530 113–116. 10.1038/nature16505 26814966 PMC4871117

[B6] Eden E. Navon R. Steinfeld I. Lipson D. Yakhini Z. (2009). GOrilla: a tool for discovery and visualization of enriched GO terms in ranked gene lists. *BMC Bioinformatics* 10:48. 10.1186/1471-2105-10-48 19192299 PMC2644678

[B7] Gaspar-Maia A. Alajem A. Polesso F. Sridharan R. Mason M. J. Heidersbach A. (2009). Chd1 regulates open chromatin and pluripotency of embryonic stem cells. *Nature* 460 863–868. 10.1038/nature08212 19587682 PMC3891576

[B8] Grotewiel M. S. Martin I. Bhandari P. Cook-Wiens E. (2005). Functional senescence in *Drosophila melanogaster*. *Ageing Res. Rev.* 4 372–397. 10.1016/j.arr.2005.04.001 16024299

[B9] Guzman-Ayala M. Sachs M. Koh F. M. Onodera C. Bulut-Karslioglu A. Lin C.-J. (2015). Chd1 is essential for the high transcriptional output and rapid growth of the mouse epiblast. *Development (Cambridge, England)* 142 118–127. 10.1242/dev.114843 25480920 PMC4299150

[B10] He Z. Luo Y. Shang X. Sun J. S. Carlson J. R. (2019). Chemosensory sensilla of the *Drosophila* wing express a candidate ionotropic pheromone receptor. *PLoS Biol.* 17:e2006619. 10.1371/journal.pbio.2006619 31112532 PMC6528970

[B11] Ismail M. Z. B. H. Hodges M. D. Boylan M. Achall R. Shirras A. Broughton S. J. (2015). The *Drosophila* insulin receptor independently modulates lifespan and locomotor senescence. *PLoS One* 10:e0125312. 10.1371/journal.pone.0125312 26020640 PMC4447345

[B12] Kari V. Mansour W. Y. Raul S. K. Baumgart S. J. Mund A. Grade M. (2016). Loss of CHD1 causes DNA repair defects and enhances prostate cancer therapeutic responsiveness. *EMBO Rep.* 17 1609–1623. 10.15252/embr.201642352 27596623 PMC5090703

[B13] Kolde R. (2019). *“pheatmap: Pretty Heatmaps”*(RRID:SCR_016418)

[B14] Konev A. Y. Tribus M. Park S. Y. Podhraski V. Lim C. Y. Emelyanov A. V. (2007). CHD1 motor protein is required for deposition of histone variant H3.3 into chromatin in vivo. *Science (New York, N.Y.)* 317 1087–1090. 10.1126/science.1145339 17717186 PMC3014568

[B15] Lee Y. Park D. Iyer V. R. (2017). The ATP-dependent chromatin remodeler Chd1 is recruited by transcription elongation factors and maintains H3K4me3/H3K36me3 domains at actively transcribed and spliced genes. *Nucleic Acids Res.* 45 7180–7190. 10.1093/nar/gkx321 28460001 PMC5499586

[B16] Loppin B. Bonnefoy E. Anselme C. Laurençon A. Karr T. L. Couble P. (2005). The histone H3.3 chaperone HIRA is essential for chromatin assembly in the male pronucleus. *Nature* 437 1386–1390. 10.1038/nature04059 16251970

[B17] Lusser A. Urwin D. L. Kadonaga J. T. (2005). Distinct activities of CHD1 and ACF in ATP-dependent chromatin assembly. *Nat. Struct. Mol. Biol.* 12 160–166. 10.1038/nsmb884 15643425

[B18] Marfella C. G. A. Imbalzano A. N. (2007). The Chd family of chromatin remodelers. *Mut. Res.* 618 30–40. 10.1016/j.mrfmmm.2006.07.012 17350655 PMC1899158

[B19] Morettini S. Tribus M. Zeilner A. Sebald J. Campo-Fernandez B. Scheran G. (2011). The chromodomains of CHD1 are critical for enzymatic activity but less important for chromatin localization. *Nucleic Acids Res.* 39 3103–3115. 10.1093/nar/gkq1298 21177652 PMC3082874

[B20] Petesch S. J. Lis J. T. (2008). Rapid, transcription-independent loss of nucleosomes over a large chromatin domain at Hsp70 loci. *Cell* 134 74–84. 10.1016/j.cell.2008.05.029 18614012 PMC2527511

[B21] Piatti P. Lim C. Y. Nat R. Villunger A. Geley S. Shue Y. T. (2015). Embryonic stem cell differentiation requires full length Chd1. *Sci. Rep.* 5:8007. 10.1038/srep08007 25620209 PMC4306112

[B22] Qi W. Yang Z. Lin Z. Park J. Y. Suh G. S. Wang L. (2015). A quantitative feeding assay in adult *Drosophila* reveals rapid modulation of food ingestion by its nutritional value. *Mol. Brain* 8:87. 10.1186/s13041-015-0179-x 26692189 PMC4687088

[B23] Rüthemann P. Balbo Pogliano C. Codilupi T. Garajovà Z. Naegeli H. (2017). Chromatin remodeler CHD1 promotes XPC-to-TFIIH handover of nucleosomal UV lesions in nucleotide excision repair. *EMBO J.* 36 3372–3386. 10.15252/embj.201695742 29018037 PMC5686551

[B24] Scheuermann E. A. Smith D. P. (2019). Odor-specific deactivation defects in a *Drosophila* odorant-binding protein mutant. *Genetics* 213 897–909. 10.1534/genetics.119.302629 31492805 PMC6827369

[B25] Schoberleitner I. Bauer I. Huang A. Andreyeva E. N. Sebald J. Pascher K. (2021). CHD1 controls H3.3 incorporation in adult brain chromatin to maintain metabolic homeostasis and normal lifespan. *Cell Rep.* 37:109769. 10.1016/j.celrep.2021.109769 34610319 PMC8607513

[B26] Schoberleitner I. Mutti A. Sah A. Wille A. Gimeno-Valiente F. Piatti P. (2019). Role for chromatin remodeling factor Chd1 in learning and memory. *Front. Mol. Neurosci.* 12:3. 10.3389/fnmol.2019.00003 30728766 PMC6351481

[B27] Sebald J. Willi M. Schoberleitner I. Krogsdam A. Orth-Höller D. Trajanoski Z. (2016). Impact of the chromatin remodeling factor CHD1 on gut microbiome composition of *Drosophila melanogaster*. *PLoS One* 11:e0153476. 10.1371/journal.pone.0153476 27093431 PMC4836739

[B28] Shenoy T. R. Boysen G. Wang M. Y. Xu Q. Z. Guo W. Koh F. M. (2017). CHD1 loss sensitizes prostate cancer to DNA damaging therapy by promoting error-prone double-strand break repair. *Ann. Oncol.* 28 1495–1507. 10.1093/annonc/mdx165 28383660 PMC5834074

[B29] Shiraiwa T. Carlson J. R. (2007). Proboscis extension response (PER) assay in *Drosophila*. *J. Vis. Exp.* 193. 10.3791/193 [Epub ahead of print]. 18978998 PMC2535836

[B30] Siggens L. Cordeddu L. Rönnerblad M. Lennartsson A. Ekwall K. (2015). Transcription-coupled recruitment of human CHD1 and CHD2 influences chromatin accessibility and histone H3 and H3.3 occupancy at active chromatin regions. *Epigenetics Chromatin* 8:4. 10.1186/1756-8935-8-4 25621013 PMC4305392

[B31] Simic R. Lindstrom D. L. Tran H. G. Roinick K. L. Costa P. J. Johnson A. D. (2003). Chromatin remodeling protein Chd1 interacts with transcription elongation factors and localizes to transcribed genes. *EMBO J.* 22 1846–1856. 10.1093/emboj/cdg179 12682017 PMC154471

[B32] Simsiii R. Millhouse S. Chen C. Lewis B. Erdjumentbromage H. Tempst P. (2007). Recognition of trimethylated histone H3 lysine 4 facilitates the recruitment of transcription postinitiation factors and Pre-mRNA splicing. *Mol. Cell* 28 665–676. 10.1016/j.molcel.2007.11.010 18042460 PMC2276655

[B33] Skene P. J. Hernandez A. E. Groudine M. Henikoff S. (2009). The nucleosomal barrier to promoter escape by RNA polymerase II is overcome by the chromatin remodeler Chd1. *Elife* 3:e02042. 10.7554/eLife.02042 24737864 PMC3983905

[B34] Slone J. Daniels J. Amrein H. (2007). Sugar receptors in *Drosophila*. *Curr. Biol.* 17 1809–1816. 10.1016/j.cub.2007.09.027 17919910 PMC2078200

[B35] Soibam B. Mann M. Liu L. Tran J. Lobaina M. Kang Y. Y. (2012). Open-field arena boundary is a primary object of exploration for *Drosophila*. *Brain Behav.* 2 97–108. 10.1002/brb3.36 22574279 PMC3345355

[B36] Srinivasan S. Dorighi K. M. Tamkun J. W. (2008). *Drosophila* Kismet regulates histone H3 lysine 27 methylation and early elongation by RNA polymerase II. *PLoS Genet.* 4:e1000217. 10.1371/journal.pgen.1000217 18846226 PMC2563034

[B37] Swarup S. Williams T. I. Anholt R. R. (2011). Functional dissection of Odorant binding protein genes in *Drosophila melanogaster*. *Genes Brain Behav.* 10 648–657. 10.1111/j.1601-183X.2011.00704.x 21605338 PMC3150612

[B38] Talbert P. B. Henikoff S. (2010). Histone variants — ancient wrap artists of the epigenome. *Nat. Rev. Mol. Cell Biol.* 11 264–275. 10.1038/nrm2861 20197778

[B39] Vang L. L. Medvedev A. V. Adler J. (2012). Simple ways to measure behavioral responses of *Drosophila* to stimuli and use of these methods to characterize a novel mutant. *PLoS One* 7:e37495. 10.1371/journal.pone.0037495 22649531 PMC3359294

[B40] Wang Z. Singhvi A. Kong P. Scott K. (2004). Taste representations in the *Drosophila* brain. *Cell* 117 981–991. 10.1016/j.cell.2004.06.011 15210117

[B41] Xiao S. Sun J. S. Carlson J. R. (2019). Robust olfactory responses in the absence of odorant binding proteins. *Elife* 8:e51040. 10.7554/eLife.51040.020 PMC6814364 31651397

