# Supplementary material for: Regulation of sensory perception and motor abilities by brain-specific action of chromatin remodeling factor CHD1
Source: Front Mol Neurosci. 2022 Aug 2;15:840966. doi: 10.3389/fnmol.2022.840966 (PMC9378821; doi:10.3389/fnmol.2022.840966)
Supplement: Supplementary file 1 [file Image_1.pdf]

Supplementary Material

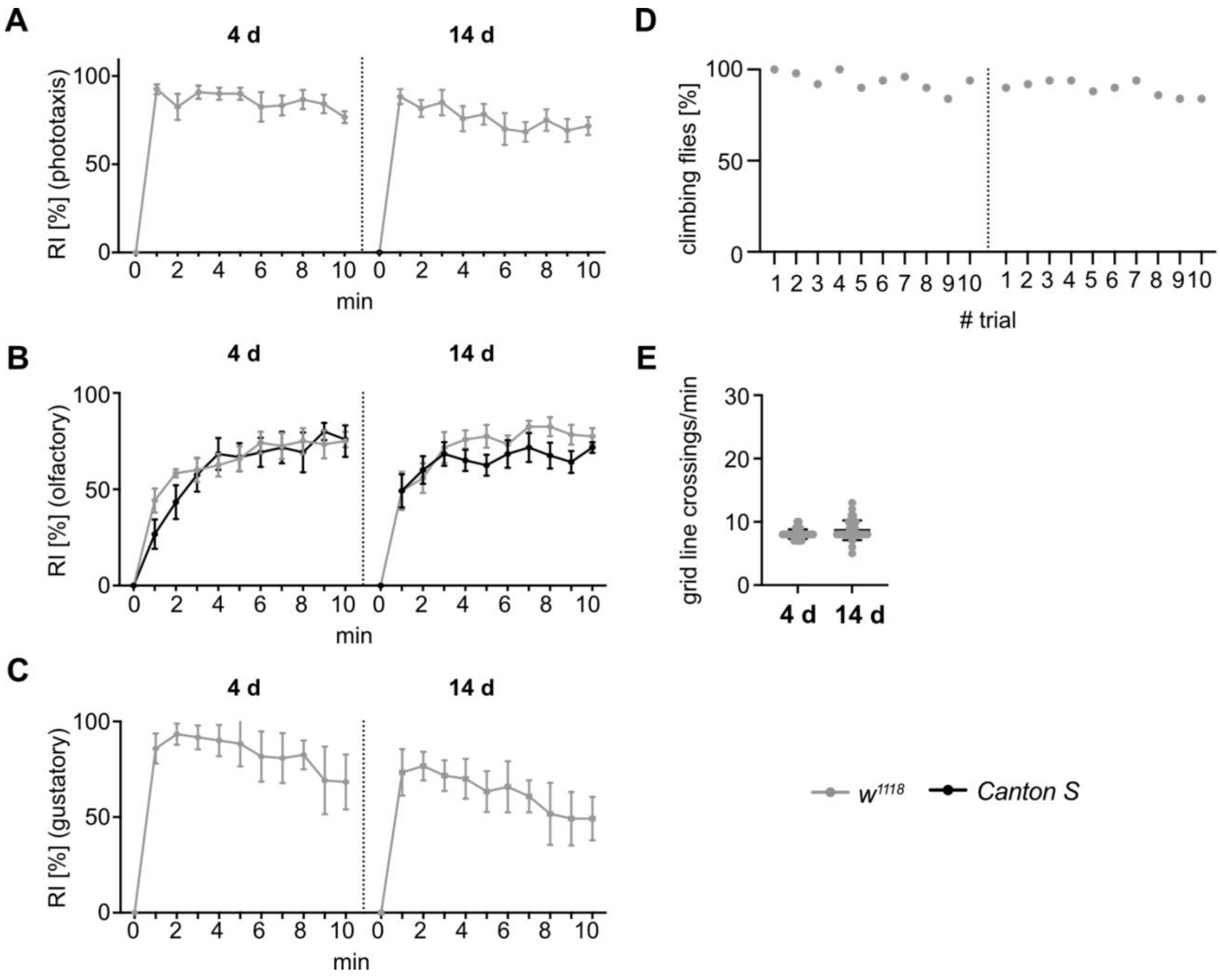

Supplementary Figure 1. Behavior of wildtype *Drosophila melanogaster* in the behavioral assays used in this study. (**A**) Positive phototaxis response indices (RI) of 4 and 14 d old w^{1118} flies. (**B**) Repulsion response indices (RI) of 4 and 14 d old w^{1118} (grey symbols) and *Canton S* (black symbols) flies to 10 mM benzaldehyde. (**C**) Attraction response indices (RI) to sucrose. The assays shown in **A-C** were performed as described in Figure 2. Mean ± SEM of six technical replicates is shown. (**D**) Percentage of 4 and 14 d old w^{1118} flies climbing to 8 cm height within 60 s in 10 trials. Mean ± SEM of 6 cohorts of 10-12 flies per genotype from one experiment is shown. (**E**) Gridline crossings of individual (n>40) 4 and 14 d old w^{1118} flies during 1 min were scored.
